# Time to recovery from severe community-acquired pneumonia and its determinants among older adults admitted to North Wollo hospitals: A multi-centred cohort study

**DOI:** 10.7189/jogh.14.04203

**Published:** 2024-09-27

**Authors:** Tegene Atamenta Kitaw, Molla Azmeraw, Dessie Temesgen, Ribka Nigatu Haile

**Affiliations:** Department of Nursing, College of Health Science, Woldia University, Woldia, Ethiopia

## Abstract

**Background:**

Severe community-acquired pneumonia presents a looming threat to older adults globally, often resulting in alarming mortality rates. Despite advancements in treatment, challenges persist, exacerbated by factors like increasing comorbidity. As age rises, so does the risk of mortality and prolonged recovery periods. Particularly in low-income countries such as Ethiopia, the burden of severe community-acquired pneumonia is staggering. Yet, research on the estimated time to recovery and its determinants among older adults in this region remains insufficient, demanding urgent attention. Hence, in this study we endeavour to uncover insights into the recovery time and contributing factors among older adults.

**Methods:**

We conducted a multi-centred retrospective cohort study among 422 older adults aged >65 years. We collected data using a structured checklist, and the final sample was meticulously selected using a systematic sampling technique. We computed Kaplan-Meier survival curves and log-rank tests to compare survival curves. We assessed multicollinearity using variance inflation factors. Further, we employed a Cox regression model to identify significant determinants, with model fitness evaluated using a Cox-Snell residual plot. Statistical significance was declared at a *P* ≤ 0.05.

**Results:**

In this study, 79.3% (95% confidence interval (CI) = 75.58–83.29) of patients achieved recovery, with a median time to recovery from severe community-acquired pneumonia of 19 days. Age >75 years, diabetes mellitus, chronic obstructive pulmonary disease, elevated creatinine level and baseline white blood cells greater than 11.0 × 10^9^/L were found to be significant determinants.

**Conclusions:**

On average, older adults take 19 days to recover from severe community-acquired pneumonia. Recovery times are notably longer for individuals aged >75 years, those with comorbidities, and those with elevated white blood cell and creatinine levels. Therefore, tailored interventions addressing these specific factors could potentially improve patient outcomes.

Severe community-acquired pneumonia (SCAP) is a major cause of morbidity and mortality, particularly among older adults. The ageing population is at increased risk due to factors such as weakened immune systems, the presence of comorbidities, and often delayed access to medical care [1–4]. SCAP is defined as the presence of severe acute respiratory failure needing invasive mechanical ventilation and/or septic shock with organ system dysfunction [5].

Lower respiratory tract infections, including community-acquired pneumonia (CAP), are the leading cause of infectious mortality globally, responsible for 6.1% of all deaths, with 2.8 million fatalities and a loss of 115 million disability-adjusted life years annually [6]. In low-income countries, CAP-related mortality is notably higher, with reported rates of 23% in Cambodia, 19% in Senegal, 18% in Uganda, 16% in the Central African Republic, and 14.6% in Malawi [7]. In sub-Saharan Africa, approximately four million cases of pneumonia occur annually, leading to over 200 000 deaths [8]. In Ethiopia, the CAP mortality rate among admitted patients is 20.2%. Patients aged >65 years, respiratory rate >30 breaths per minute, and comorbid tuberculosis were associated with poor treatment outcomes. The mean duration of hospital stay following infection with CAP is 11.49 days [9].

Despite medical advancements, CAP remains deadly, with 20% of patients requiring hospitalisation and a quarter of severe cases needing invasive mechanical ventilation [10]. Older adults often face prolonged recovery, with symptoms lasting over three weeks and up to 13 days of absence from normal activities [11]. SCAP significantly impacts health-related quality of life, with a 16% decline in post-discharge quality of life among survivors [12]. The economic burden is substantial, with treatment costs around EUR 10.1 billion and lost workdays adding EUR 3.6 billion [13].

Although improvements in medical treatment have been made, recovery times from SCAP continue to vary widely due to a range of clinical and demographic factors [14,15]. Understanding these recovery times and their determinants is crucial for improving patient outcomes, particularly in resource-limited settings such as North Wollo, Ethiopia. North Wollo zone, impacted by ongoing conflict, presents unique socio-economic and health care delivery characteristics that reflect many low-resource environments. The region’s health care infrastructure faces significant challenges, including limited diagnostic and treatment resources, shortages of medical equipment, and restricted access to advanced diagnostics [16]. The ongoing conflict exacerbates these issues, further complicating health care delivery and affecting the quality of care. Additionally, older adults in this setting are experiencing increasing vulnerabilities and evolving health care needs.

With this study, we contribute to the existing literature by shedding light on the time to recovery from SCAP and its determinants among older adults, a relatively understudied area, particularly in low-resource settings like North Wollo hospitals. While much of the existing research focuses on SCAP in broader populations, our study specifically examines older adults, a group more vulnerable to prolonged recovery due to age-related physiological changes and comorbidities. Additionally, the multi-centred approach provides a more comprehensive understanding of how varying health care infrastructures and regional factors impact recovery times, addressing a significant gap in the literature that often overlooks the influence of health care setting variability. In this study, we hypothesise that specific clinical and demographic factors, including comorbidities, nutritional status, and the initial severity of pneumonia, significantly influence the time to recovery from SCAP among older adults. The primary aim of the research was to determine the time to recovery, while secondary objectives included identifying the determinants of recovery time. The research aims to highlight critical factors for intervention, inform health care strategies to optimise recovery times, and improve patient management, ultimately reducing the burden of pneumonia in similar resource-limited and conflict-affected settings.

## METHODS

### Study setting, period, and design

The study was conducted in North Wollo zone hospitals. North Wollo is in the Amhara region, with the capital city of Woldia located 521 km from Addis Ababa and 360 km from Bahirdar. There are five governmental health hospitals in North Wollo zones – Woldia Comprehensive Specialised Hospital, Kobo Primary Hospital, Lalibela Primary Hospital, Mekiet Primary Hospital, and Wadila Primary Hospital. We conducted a multi-centred retrospective cohort study from 1 August to 30 September 2023.

### Population, eligibility, and sampling technique

The source population was all older adults (≥65 years) with SCAP admitted at North Wollo hospitals. The study population consisted of all older adults (≥65 years) with severe community-acquired pneumonia admitted to selected North Wollo Zone hospitals. The inclusion criteria for the study required that all medical records of older adults aged ≥65 years who were diagnosed with SCAP during the defined follow-up period be considered for inclusion. To qualify, individuals needed to meet two primary conditions: age ≥65 years and SCAP diagnosis, as documented in their medical records. From five hospitals, we selected three hospitals (Woldia Comprehensive Specialised Hospital, Mekiet Primary Hospital, and Wadila Primary Hospital) using simple random sampling with the lottery method. An estimated 875 older patients with SCAP were considered from these hospitals. Specifically, the patient numbers were as follows: Woldia Comprehensive Specialised Hospital (n = 445), Mekiet Primary Hospital (n = 225), and Wadila Primary Hospital (n = 205). The sampling interval was calculated by dividing 875 by 422. The total estimated number of patients was proportionally allocated to each selected hospital based on patient flow. The final sample was then selected using a systematic sampling technique with a sampling interval of two. We used systematic sampling to ensure a representative and unbiased selection of records. This technique offers several advantages. It provides a straightforward and efficient method for sampling, reduces selection bias by applying a consistent procedure, and helps manage large data sets by selecting records at regular intervals. This approach ensures a comprehensive and objective analysis of the variables.

### Variables and definitions

The dependent variable is the time (in day) at which recovery from SCAP occurred. We considered different explanatory variables under socio-demographics, clinical characteristics on admission, comorbidities, treatment-related factors, and laboratory markers to identify determinants of the time needed to recover from SCAP. In this study, the event of interest referred to recovery from SCAP during the follow-up. ‘Censored’ means patients who did not develop the outcome of interest during the follow-up period (death, lost follow-up, transfer to other facilities). Incomplete patient charts represent charts that have no date of admission, discharge and outcome.

### Data extraction

We used a structured checklist to collect data. This checklist was meticulously designed to ensure comprehensive data capture. It included five main sections: section focused on basic socio-demographic variables of the patients; section gathering information on patient comorbidities; section assessing clinical characteristics at admission; section addressing laboratory findings; and section covering treatment-related factors. The checklist underwent a thorough validation process to ensure its effectiveness, including reviews by infectious diseases and geriatrics experts and a pilot test to refine its clarity and comprehensiveness. The checklist was completed by trained data collectors with a bachelor’s degree in health informatics. These professionals were specifically trained to use the checklist, medical record review, and data entry to ensure consistent and accurate application. We selected variables based on their relevance to the research objectives, including initial pneumonia severity, comorbidities, and treatment details. The choice was influenced by data availability and consistency in existing records, prior research, and practical considerations of data completeness and reliability.

### Data processing and analysis

Using the structured checklist, we meticulously coded, cleaned, and edited collected data to ensure accuracy and completeness. We employed Epi-Data, version 4.2 (EpiData Association, Odense, Denmark) for data entry, providing a robust platform for managing and organising the collected information. After data entry, we further processed the data set using Stata, version 17.0 (StataCorp LLC, College Station, Texas, USA). We addressed missing data using appropriate techniques, such as data imputation or exclusion, depending on the extent and nature of the missing information. Descriptive statistics were first calculated to summarise the data. For continuous variables, we computed measures of central tendency (such as means and medians) and dispersion (including standard deviations and ranges). We analysed categorical data using frequency distributions to capture the proportion of each category within the data set.

We generated a Kaplan-Meier survival curve to assess the time to recovery from SCAP. This curve visually represented recovery times and allowed for estimating survival probabilities over time. Differences in survival curves between different categories of variables were evaluated using the log-rank test, which helps determine whether the observed differences in survival are statistically significant. Before conducting survival analysis, we assessed multicollinearity among the predictor variables using variance inflation factors and tolerance values to ensure that predictors were not highly correlated, which could affect the stability of the regression model. We used the Cox proportional hazards regression model to identify significant determinants of recovery time from SCAP. The proportional hazards assumption was tested using Schoenfeld residuals to ensure the validity of the model. The goodness of fit for the Cox regression model was evaluated using a Cox-Snell residual plot, which assesses how well the model fits the data.

The multivariable Cox regression model included variables with a *P*-value ≤0.25 in the bivariate analysis. In this analysis, variables with a *P* ≤ 0.05 were considered statistically significant. The strength of associations between predictors and recovery time was quantified using hazard ratios with 95% confidence intervals (CIs). Finally, we presented the findings from the analysis using a combination of textual descriptions, tables, and graphs.

## RESULTS

### Sociodemographic and clinical characteristics of the participants

The median age of the participants was 71 years (interquartile range (IQR) = 68–74). The majority of participants (64.5%) were male. Concerning clinical characteristics, most patients experienced cough (96.2%) and shortness of breath (80.1%). Additionally, 46.4% of the participants reported chest pain (**Table 1**).

**Table 1:** Sociodemographic and clinical characteristics of older adults with severe community-acquired pneumonia in North Wollo Hospitals, Amhara region, Ethiopia, 2023

Variables	Censored, n (%)	Recovery, n (%)	Total, n (%)
Age in years			
*65–70*	33 (7.8)	176 (41.7)	209 (49.5)
*71–75*	31 (7.3)	117 (27.7)	148 (35.1)
*>75*	23 (5.5)	42 (10.0)	65 (15.4)
Sex			
*Male*	66 (15.6)	206 (48.8)	272 (64.5)
*Female*	21 (5.0)	129 (30.6)	150 (35.5)
Cough			
*No*	2 (0.5)	14 (3.3)	16 (3.8)
*Yes*	85 (20.1)	321 (76.1)	406 (96.2)
Fever			
*No*	8 (1.9)	79 (18.7)	87 (20.6)
*Yes*	79 (18.7)	256 (60.7)	335 (79.4)
Shortness of breath			
*No*	10 (2.4)	74 (17.5)	84 (19.9)
*Yes*	77 (18.2)	261 (61.8)	338 (80.1)
Chest pain			
*No*	28 (6.6)	194 (46.0)	222 (52.6)
*Yes*	59 (14.0)	141 (33.4)	200 (47.4)
Fatigue			
*No*	19 (4.5)	54 (12.8)	73 (17.3)
*Yes*	68 (16.1)	281 (66.6)	349 (82.7)
Headache			
*No*	33 (7.8)	104 (24.6)	137 (32.5)
*Yes*	54 (12.8)	231 (54.7)	285 (67.5)
Vomiting			
*No*	55 (13.0)	163 (38.6)	218 (51.7)
*Yes*	32 (7.6)	172 (40.8)	204 (48.3)
Loss of appetite			
*No*	86 (20.4)	326 (77.3)	412 (97.6)
*Yes*	1 (0.2)	9 (2.1)	10 (2.4)
Arthralgia			
*No*	42 (10.0)	116 (27.5)	158 (37.4)
*Yes*	45 (10.7)	219 (51.9)	264 (62.6)
Oxygen saturation			
*<95%*	76 (18.0)	264 (62.6)	340 (80.6)
*≥95%*	11 (2.6)	71 (16.8)	82 (19.4)

### Comorbidities

Nearly half of the participants (49.5%) had a comorbidity. Compared to all comorbidities, the majority of the participants (30.1%) had hypertension, followed by diabetes mellitus and congestive heart failure, 15.2% and 11.8% (**Table 2**).

**Table 2:** Comorbidity distributions of older adults with severe community-acquired pneumonia in North Wollo Hospitals, Amhara region, Ethiopia, 2023

Variables	Censored, n (%)	Recovery, n (%)	Total, n (%)
**Comorbidity**			
*No*	14 (3.3)	199 (47.2)	213 (50.5)
*Yes*	73 (17.3)	136 (32.2)	209 (49.5)
Number of comorbidities			
*1*	35 (8.3)	96 (22.7)	131 (31.0)
*≥2*	35 (8.3)	43 (10.2)	78 (18.5)
Congestive heart failure			
*No*	67 (15.9)	305 (72.3)	372 (88.2)
*Yes*	20 (4.7)	30 (7.1)	50 (11.8)
Diabetes mellitus			
*No*	51 (12.1)	307 (72.7)	358 (84.8)
*Yes*	36 (8.5)	28 (6.6)	64 (15.2)
Hypertension			
*No*	43 (10.2)	252 (59.7)	295 (69.9)
*Yes*	44 (10.4)	83 (19.7)	127 (30.1)
COPD			
*No*	39 (9.2)	294 (69.7)	333 (78.9)
*Yes*	48 (11.4)	41 (9.7)	89 (21.1)
Asthma			
*No*	85 (20.1)	322 (76.3)	407 (96.4)
*Yes*	2 (0.5)	13 (3.1)	15 (3.6)
HIV/AIDS			
*No*	84 (19.9)	327 (77.5)	411 (97.4)
*Yes*	3 (0.7)	8 (1.9)	11 (2.6)
Chronic liver disease			
*No*	85 (20.1)	327 (77.5)	412 (97.6)
*Yes*	2 (0.5)	8 (1.9)	10 (2.4)
Stroke			
*No*	77 (18.2)	326 (77.3)	403 (95.5)
*Yes*	10 (2.4)	9 (2.1)	19 (4.5)
*Chronic kidney disease*			
No	80 (19.0)	319 (75.6)	399 (94.5)
Yes	7 (1.7)	16 (3.8)	23 (5.5)

COPD – chronic obstructive pulmonary disease

### Laboratory and treatment-related characteristics

Analysis of basic laboratory markers revealed that 23.8% of the total patient population exhibited leucocytosis. Furthermore, thrombocytopenia was observed in 19.2% of the study cohort. Regarding treatment-related characteristics, it was noted that approximately 22.2% of the study sample required mechanical ventilation as part of their therapeutic management (**Table 3**).

**Table 3:** Laboratory and treatment-related characteristics of older adults with severe community-acquired pneumonia in North Wollo Hospitals, Amhara region, Ethiopia, 2023

Variables	Censored, n (%)	Recovery, n (%)	Total, n (%)
WBC (× 10^9^/L)			
*<4.5*	2 (0.5)	25 (5.9)	27 (6.4)
*4.5–11*	24 (5.7)	261 (61.8)	285 (67.5)
*>11*	61 (14.5)	49 (11.6)	110 (26.1)
Platelet count (μL)			
*<150 000*	28 (6.6)	54 (12.8)	82 (19.4)
*≥150 000*	59 (14.0)	281 (66.6)	340 (80.6)
Haemoglobin in g/dl (x̄)			
*≤14.2*	52 (12.3)	157 (37.2)	209 (49.5)
*>14.2*	35 (8.3)	178 (42.2)	213 (50.5)
Haematocrit (%)			
*≤39.8*	51 (12.1)	127 (30.1)	178 (42.2)
*>39.8*	36 (8.5)	208 (49.3)	244 (57.8)
Random blood glucose level in mg/dl			
*≤116.3*	41 (13.0)	184 (58.4)	225 (71.4)
*>116.3*	24 (7.6)	66 (21.0)	90 (28.6)
Ceftriaxone			
*No*	13 (3.1)	28 (6.6)	41 (9.7)
*Yes*	74 (17.5)	307 (72.7)	381 (90.3)
Vancomycin			
*No*	48 (11.4)	306 (72.5)	354 (83.9)
*Yes*	39 (9.2)	29 (6.9)	68 (16.1)
Mechanical ventilation			
*No*	45 (10.7)	282 (66.8)	327 (77.5)
*Yes*	42 (10.0)	53 (12.6)	95 (22.5)

WBC – white blood cells, x̄ – mean

### Survival time to recovery from SCAP

**Figure 1:**
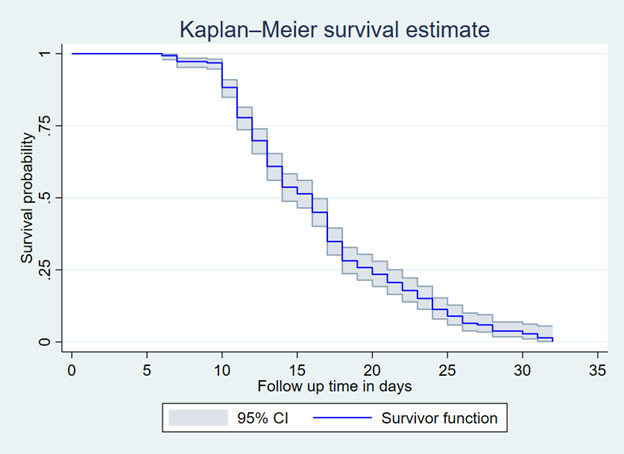
Overall Kaplan-Meier survival curve of time to recovery from severe community-acquired pneumonia in North Wollo Hospitals, Amhara region, Ethiopia, 2023.

In this study, throughout the follow-up period, 79.3% (95% CI = 75.58–83.29) of patients recovered from SCAP. The overall median survival time to recover from SCAP was 19 days (IQR = 16–22). The total follow-up time contributed by all study participants was 6886 per day of observation (**Figure 1**).

### Comparisons of survival functions of different categorical variables

We computed the Kaplan-Meier survival curve and the Log-rank test to compare and estimate the survivor function among different groups of variables. In the Kaplan-Meier survival curve, one survivorship function curve located under another means the lower curve group has a lower survival status than the upper curve group or has a less desirable survival probability than the upper curve. Furthermore, the difference was described statistically by the log-rank test.

**Figure 2:**
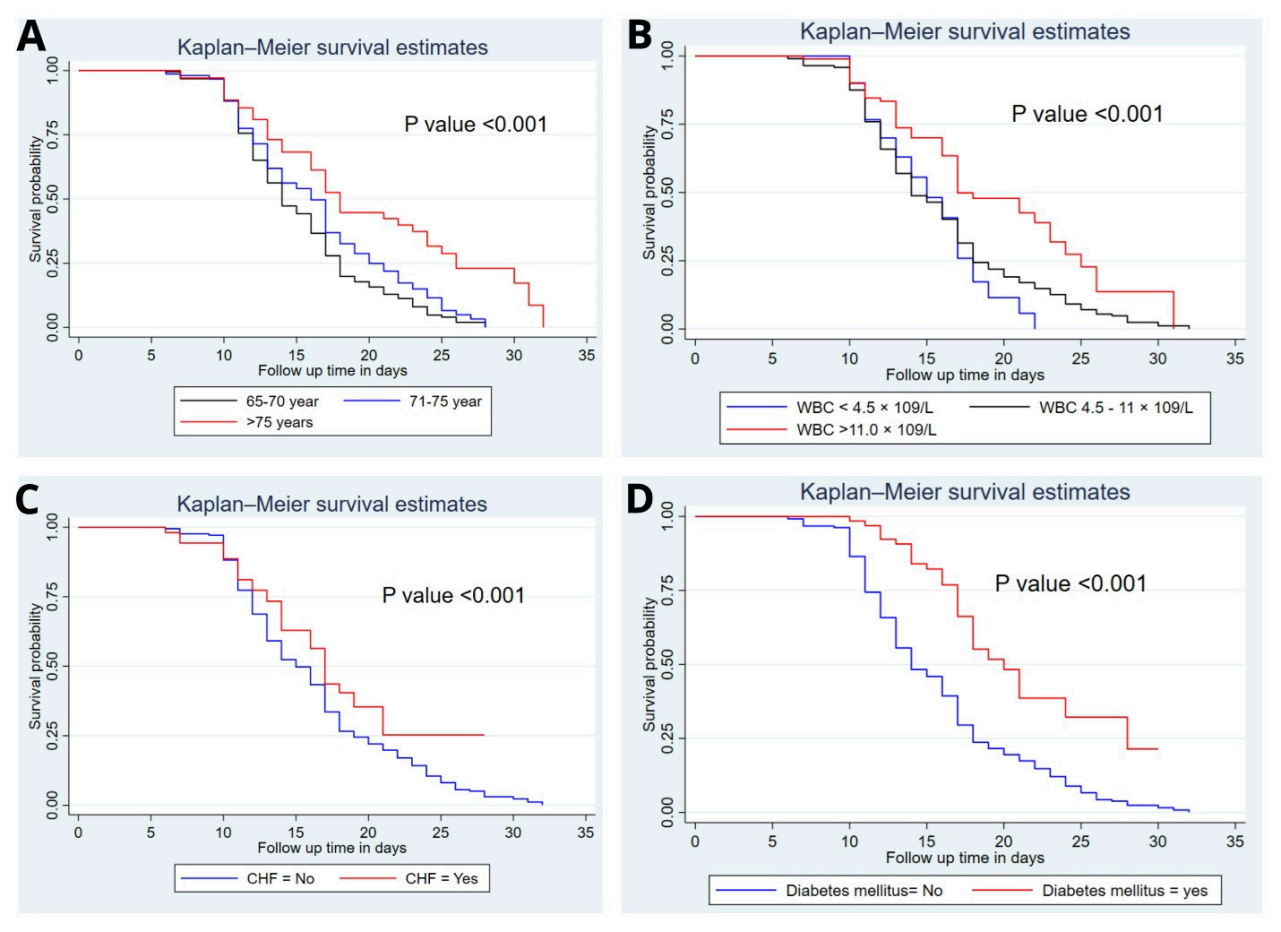
Kaplan-Meier survival curves and log-rank tests among older adults with severe community-acquired pneumonia in North Wollo Hospitals, Amhara region, Ethiopia, 2023. **Panel A.** Age difference. **Panel B.** Baseline white blood cell levels. **Panel C.** Congestive heart failure. **Panel D.** Diabetes mellitus.

Generally, the Kaplan-Meier survival curve showed that older adults with SCAP who are aged >75 years have a baseline white blood cells (WBC) >11.0 × 10^9^/L, and the presence of comorbidity, mainly congestive heart failure and diabetes mellitus takes more time to recover than the reverse group (**Figure 2**).

### Proportional hazard assumption test

The proportional hazard assumption test by Schoenfeld residual revealed that the ρ statistic *P*-value of all covariates is >0.05, and the global test *P* = 0.1379 (>0.05). This means that the Cox- proportional hazard assumption is satisfied (Table S1 in the **Online Supplementary Document**).

### The goodness of fit of the Cox regression model

**Figure 3:**
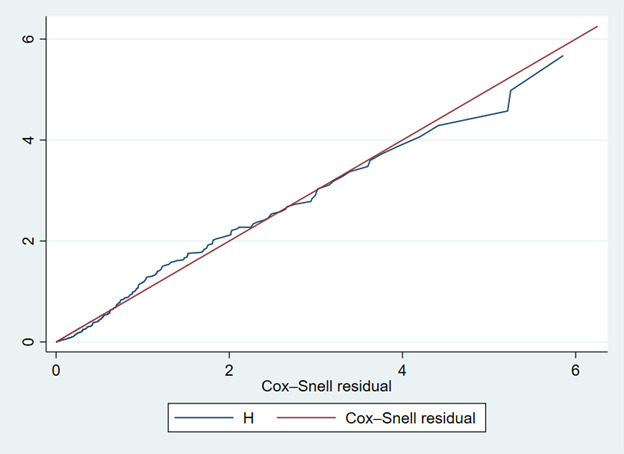
Cox-Snell residual plot to test the goodness of fit of the Cox regression model.

The goodness of fit for the Cox regression model was assessed using a Cox-Snell residual plot. Analysis of the plot revealed that the residuals align closely with the reference line (within a 45° alignment), indicating a strong fit between the model and the data (**Figure 3**).

### Determinants of time to recovery from SCAP

In bivariate Cox proportional-hazards analysis, age, sex, chest pain at admission, shortness of breathing, comorbidity, congestive heart failure, diabetes mellitus, chronic obstructive pulmonary disease (COPD), numbers of comorbidity, WBC, creatinine level, ceftriaxone and vancomycin were found to be statistically significant (*P* < 0.25). In multivariant analysis, age, comorbidity, diabetes mellitus, COPD, creatinine level, and WBC were significant determinants of time to recovery from severe community-acquired pneumonia.

Older adults aged >75 years experienced a 54% longer recovery time than those aged between 65–70 years (adjusted hazard ratio (AHR) = 0.46; 95% CI = 0.32–0.65). Older adults with elevated creatinine levels were 49% more likely to have a delayed recovery from SCAP than those with normal levels (AHR = 0.51; 95% CI = 0.30–0.86). The hazard of recovery time from SCAP was 36% longer among older adults with comorbidities than those without (AHR = 0.64; 95% CI = 0.46–0.88). Specifically, older adults with diabetes mellitus and COPD had a 47% (AHR = 0.53; 95% CI = 0.35–0.81) and 49% (AHR = 0.51; 95% CI = 0.37–0.69) longer recovery time compared to those without these conditions. Additionally, a baseline WBC count greater than 11.0 × 10^9^/L was associated with a 41% longer recovery time compared to those with WBC counts within the normal range (AHR = 0.59; 95% CI = 0.43–0.82) (**Table 4**).

**Table 4:** Cox regression analysis for determinants of time to recovery from severe community-acquired pneumonia among older adults in North Wollo Hospitals, Amhara region, Ethiopia, 2023

Variables	CHR (95% CI)	AHR (95% CI)	*P*-value
Age in years			
*65–70*	ref.	ref.	
*71–75*	0.75 (0.59–0.95)	0.81 (0.65–1.03)	0.082
*>75*	0.38 (0.27–0.52)	0.46 (0.32–0.65)	0.000*
Sex			
*Male*	ref.	ref.	
*Female*	1.03 (0.83–1.27)	1.03 (0.80–1.26)	0.973
Chest pain at admission			
*No*	ref.	ref.	
*Yes*	0.64 (0.52–0.79)	0.66 (0.53–1.09)	0.098
Shortness of breathing at admission			
*No*	ref.	ref.	
*Yes*	0.74 (0.52–1.31)	0.81 (0.68–1.42)	0.092
Comorbidity			
*No*	ref.	ref.	
*Yes*	0.45 (0.34–0.58)	0.64 (0.46–0.88)	0.007†
Congestive heart failure			
*No*	ref.	ref.	
*Yes*	0.76 (0.53–1.10)	0.87 (0.75–1.78)	0.501
Diabetes mellitus			
*No*	ref.	ref.	
*Yes*	0.39 (0.27–0.57)	0.53 (0.35–0.81)	0.004†
COPD			
*No*	ref.	ref.	
*Yes*	0.40 (0.29–0.54)	0.51 (0.37–0.69)	0.000*
Number of comorbidities			
*0*	ref.	ref.	
*1*	0.66 (0.52–0.84)	1.09 (0.83–1.45)	0.529
*≥2*	0.46 (0.33–0.64)	1.13 (0.74–1.72)	0.568
WBC ( × 10^9^/L)			
*<4.5*	0.75 (0.61–1.04)	0.81 (0.68–1.26)	0.098
*4.5–11*	ref.	ref.	Ref.
*>11*	0.41 (0.30–0.55)	0.59 (0.43–0.82)	0.002†
Creatinine in mg/dL			
*<0.5*	0.78 (0.52–1.24)	0.81 (0.47–1.38)	0.438
*0.5–1.5*	ref.	ref.	
*>1.5*	0.43 (0.28–0.75)	0.51 (0.30–0.86)	0.012‡
Ceftriaxone			
*No*	ref.	ref.	
*Yes*	1.05 (0.74–1.51)	1.23 (0.82–1.77)	0.504
Vancomycin			
*No*	ref.	ref.	
*Yes*	1.34 (0.89–1.75)	1.62 (0.92–2.53)	0.068

AHR – adjusted hazard ratio, CHR – crude hazard ratio, CI – confidence interval, COPD – chronic obstructive pulmonary disease, ref – reference, WBC – white blood cells

*Statistical significance at *P* = 0.001.

†Statistical significance at *P* = 0.01.

‡Statistical significance at *P* = 0.05.

## DISCUSSION

In this study, 79.3% of patients recovered, with a median time to recovery from SCAP of 19 days. Age >75 years, comorbidity, diabetes mellitus, COPD, elevated creatinine level, and baseline WBC>11.0 × 10^9^/L were found to be significant determinants of time to recovery from SCAP.

The finding of the study is lower than that reported in other studies conducted in Ethiopia (87.3%) [17], Kenya (90%) [18], and Canada (93.2%) [19]. However, our findings are higher than in another study conducted in Ethiopia, where the recovery rate was reported to be 56% [20]. Interestingly, our study aligns with the research results conducted at Jimma University Specialised Hospital, which reported a prevalence of 79.8% [9]. The variation in recovery rates observed across different studies can be attributed to several factors, including differences in study populations, health care infrastructure, and treatment practices. For instance, disparities in patient demographics, illness severity, and comorbidities may influence recovery outcomes. Variations in health care resources, access to care, and methodological approaches, such as study design and data collection methods, may also contribute to the discrepancies. Furthermore, differences in health care practices, treatment protocols, and cultural beliefs could significantly impact recovery rates.

Compared to previous studies where the clinical cure was achieved at 28 days [21] and 21 days [11], the recovery time observed in our study is shorter. The variation could be due to recent advancements in treatment and technology that significantly contribute to timely recovery from SCAP. Advancements in treatment may include the development of more effective antibiotics targeting the specific pathogens responsible for SCAP and introducing novel therapeutic approaches such as immunomodulatory drugs or adjunctive therapies. Additionally, improvements in supportive care measures, including respiratory support and fluid management, could enhance recovery outcomes. Furthermore, patient characteristics, disease severity, treatment protocols, health care infrastructure, timing of the studies, and environmental factors might also contribute to the differences.

This finding aligns with previous research [22,23], showing that individuals aged >65 years experience prolonged recovery and poorer outcomes from SCAP. Contributing factors include age-related declines in immune function, a higher prevalence of underlying health conditions, reduced physiological reserves, increased susceptibility to complications, and the potential effects of polypharmacy. The weakened immune response and diminished physiological function in older adults impair their ability to combat infections effectively [23–25]. Therefore, close monitoring and appropriate interventions are essential to support recovery and improve outcomes in this vulnerable population.

Previous studies [20,24] also confirm that elevated creatinine levels impact recovery from SCAP. Elevated creatinine levels can result from dehydration due to high fever, increased respiratory rate, and reduced fluid intake, which impair kidney function and creatinine clearance [25,26]. Monitoring creatinine levels in elderly patients with SCAP is essential for anticipating longer recovery times and planning appropriate management strategies. Early detection can prompt closer monitoring and more aggressive interventions to prevent complications and improve outcomes in this vulnerable population.

The presence of underlying health conditions complicates the recovery process from SCAP, as evident in other studies [17]. Diabetes mellitus weakens the immune system, reducing the ability to fight infections, while COPD exacerbates respiratory distress. Chronic inflammation and metabolic dysfunction in these conditions delay tissue repair and intensify inflammatory responses [27,28]. Additionally, age-related physiological changes and the need for careful medication management add complexity to treatment. This suggests that the presence of underlying health conditions complicates the recovery process from SCAP, possibly due to factors such as compromised immune function, reduced physiological reserves, or increased susceptibility to complications. The prolonged recovery times in severe pneumonia patients with comorbidities like diabetes mellitus and COPD are influenced by several factors. Diabetes mellitus weakens the immune system, making it harder to fight infections effectively, while COPD worsens respiratory distress, complicating lung function further. Chronic inflammation and metabolic dysfunction in both conditions delay tissue repair and exacerbate inflammatory responses. Other comorbidities and age-related physiological changes add complexity to treatment, as does the need for careful medication management. These findings underscore the need for health policies that prioritise early intervention and comprehensive management strategies for patients with SCAP who have underlying comorbidities.

A baseline increase in WBC count from normal ranges is associated with delayed recovery time, as other studies have also concluded that leucocytosis is linked to prolonged recovery and unfavourable outcomes [20,29]. Elevated WBC levels, particularly leucocytosis, suggest that the body is fighting a significant infection, which can overwhelm the immune system and impair the body's ability to resolve the infection quickly. This implies the importance of monitoring WBC count at SCAP onset for prognostic purposes. Patients with elevated WBC counts may require closer monitoring, aggressive treatment, or extended hospital stays. Addressing underlying conditions contributing to high WBC levels could expedite recovery. Overall, tailored management that considers baseline WBC count is crucial for optimising recovery in elderly SCAP patients.

The multi-centred design of the study is a notable strength, as it allows for broader generalizability of the findings beyond individual health care facilities. By including multiple centres, the study captures a more diverse patient population, increasing the robustness and applicability of the results to different clinical contexts. However, the retrospective nature of the study poses certain limitations. Relying on retrospective data may hinder the exploration of additional factors influencing recovery time from SACP. Incomplete or inaccurately recorded information could affect the validity of the findings, while missing data may impact the reliability and generalizability of the results. Furthermore, the absence of patient and public input may limit the relevance of our findings to the broader population, as we did not capture perspectives that could have informed the study design, data interpretation, or contextual relevance.

## CONCLUSIONS

We found that the median recovery time from SCAP is 19 days. Significant factors influencing recovery included age >75 years, comorbidities, diabetes mellitus, COPD, a baseline WBC>11.0 × 10^9^/L, and elevated creatinine levels. Therefore, it is recommended that triage protocols be developed and integrated to identify patients with high-risk factors (eg, advanced age, comorbidities) upon admission. This will enable prioritised and tailored interventions to accelerate recovery. For patients with elevated baseline WBC counts and creatinine levels, establish specific treatment pathways that include early initiation of advanced therapies and close monitoring, with routine reassessment of these markers to adjust treatment plans as needed. Create comprehensive management plans for patients with diabetes mellitus and COPD, incorporating regular reviews of these conditions and adjusting their management to support better SCAP recovery. Consider establishing dedicated clinics or care teams to address these needs. Form specialised multidisciplinary teams, including pulmonologists, infectious disease specialists, and diabetes educators, to manage SCAP patients with comorbid conditions. Ensure these teams collaborate on individualised patient care plans to optimise treatment outcomes.

## Additional material


Online Supplementary Document
