# Supplementary material for: Time to recovery from severe community-acquired pneumonia and its determinants among older adults admitted to North Wollo hospitals: A multi-centred cohort study
Source: J Glob Health. 2024 Sep 27;14:04203. doi: 10.7189/jogh.14.04203 (PMC11426932; doi:10.7189/jogh.14.04203)
Supplement: Online Supplementary Document [file jogh-14-04203-s001.pdf]

Schoenfeld residuals tests

Table S1:Results of assumptions for the Cox proportional hazards model using Schoenfeld residuals tests.

Variables	rho	chi2	df	Prob>chi2
Sex	-0.00562	0.01	1	0.9134
Age	-0.05218	0.97	1	0.3248
Chest pain	0.25885	22.80	1	0.1007
Oxygen saturation	0.01790	0.11	1	0.7390
Congestive heart failure	0.02228	0.19	1	0.6602
Diabetes mellites	0.12091	5.39	1	0.1203
COPD	0.15167	7.97	1	0.1047
Comorbidity	0.03000	0.36	1	0.5476
Number of comorbidities	-0.05087	0.89	1	0.3463
White blood cell count	0.01036	0.04	1	0.8466
Ceftriaxone	-0.02017	0.14	1	0.7084
Vancomycin	-0.19818	18.12	1	0.2091
Global test		55.69	12	0.1379
